# Case Report: VEXAS syndrome with extensive pulmonary, cardiac, and skeletal involvement

**DOI:** 10.3389/fimmu.2025.1724045

**Published:** 2025-11-28

**Authors:** Zhongbiao Fang, Tingwei Xu, Meng Zhang, Chaonan Li, Anwen Zheng, Yudan Gu, Chuhan Lan, Zongming Liu, Lingling Tang

**Affiliations:** 1 Key Laboratory of Artificial Organs and Computational Medicine in Zhejiang Province, Shulan (Hangzhou) Hospital, Shulan International Medical College, Zhejiang Shuren University, Hangzhou, China; 2 School of Medicine, Zhejiang Chinese Medical University, Shuren College, Hangzhou, China; 3 The First Affiliated Hospital, School of Medicine, Zhejiang University, Hangzhou, China; 4 Clinical Medical College of Hangzhou Normal University, Hangzhou, China; 5 Emory College of Arts and Sciences, Emory University, Atlanta, GA, United States; 6 Department of Infectious Diseases, Shulan (Hangzhou) Hospital, Shulan International Medical College, Zhejiang Shuren University, Hangzhou, China; 7 Department of laboratory medicine, Hangzhou Tongchuang Medical Laboratory, Hangzhou, China

**Keywords:** VEXAS syndrome, uba1, autoinflammatory disease, vasculitis, case report

## Abstract

VEXAS syndrome is a rare and severe systemic inflammatory disorder caused by somatic mutations in the X-linked UBA1 gene, primarily affecting men. Since its initial description in 2020, it has been recognized for its complex clinical phenotype and tendency to be misdiagnosed. We report a case of a 77-year-old Chinese man diagnosed with VEXAS syndrome. The patient presented with recurrent fever, elevated inflammatory markers, anemia (decreased hemoglobin), multifocal interstitial pneumonia, and cardiac arrhythmia. On the day of admission, the patient developed rapidly progressive respiratory distress with a marked worsening of inflammatory markers. While providing supportive symptomatic treatment, we performed next-generation sequencing (NGS), 18F-fluorodeoxyglucose positron emission tomograph–computed tomography (18FDG PET-CT), and whole-exome sequencing. Based on a presumed clinical diagnosis of small-vessel vasculitis, the patient was empirically treated with glucocorticoids combined with intravenous immunoglobulin (IVIG). Once the patient’s condition improved, whole-exome sequencing revealed a UBA1 splice-site mutation (c.118-1G>C), consistent with VEXAS syndrome. After reviewing related reports, we subsequently performed a bone marrow aspiration, which showed characteristic cytoplasmic vacuolization in myeloid precursor cells. Retrospective history review revealed that the patient had developed skin lesions one year before the onset of fever. The clinical presentation of VEXAS syndrome is heterogeneous and associated with high mortality. It can be difficult to distinguish VEXAS from other autoimmune diseases, hematologic malignancies, and infectious diseases. In this case, given the patient’s rapidly progressive interstitial pneumonia, we used NGS and 18FDG PET-CT to exclude infection and hematologic malignancy, and focused on empirical treatment for presumed small-vessel vasculitis, which quickly halted disease progression. Meanwhile, whole-exome sequencing ultimately identified the underlying cause.

## Introduction

The name “VEXAS” is derived from five key features (the initial letters of each word): V (vacuoles, predominantly seen in myeloid and erythroid precursor cells), E (E1 enzyme, referring to the ubiquitin-activating enzyme encoded by UBA1), X (X-linked gene), A (autoinflammatory), and S (somatic, indicating somatic rather than germline mutations). In 2020, Beck et al. (1) reported that 25 male patients with systemic inflammation and/or hematologic disease had somatic mutations in the UBA1 gene (ubiquitin-like modifier-activating enzyme 1) in hematopoietic progenitor cells, identified by Sanger sequencing, and found that this mutation is associated with VEXAS syndrome. The clinical presentation of VEXAS syndrome is that of an adult-onset, treatment-refractory inflammatory syndrome caused by morphological and functional abnormalities of hematopoietic and myeloid precursor cells. The inflammation can involve multiple organs, manifesting as recurrent fever, rash, relapsing polychondritis (auricular/nasal chondritis), vasculitis, pulmonary inflammation, myelodysplastic syndrome, thrombotic events, and other features (1, 2).

There is currently no definitive treatment for VEXAS syndrome. Given its pathogenesis involving excessive pro-inflammatory cytokine production and chronic systemic inflammation, the main therapeutic strategies include eradicating the UBA1-mutant hematopoietic clone (e.g., via allogeneic hematopoietic stem cell transplantation) and blocking the inflammatory cascade (e.g., high-dose glucocorticoids, JAK inhibitors, or IL-1 receptor antagonists) (3–6). Management of complications is also important, including red blood cell and platelet transfusions, use of erythropoiesis-stimulating agents, prophylactic anticoagulation, and prophylactic antibiotics (5).

Here, we present an elderly male patient who experienced recurrent fever, pulmonary inflammation, and arrhythmia. When his disease rapidly accelerated, glucocorticoid therapy was used to control disease progression. At the same time, rapid high-throughput diagnostic methods including NGS, 18FDG PET-CT, and whole-exome sequencing were employed, ultimately confirming a diagnosis of VEXAS syndrome.

## Case description

**Figure 1:**
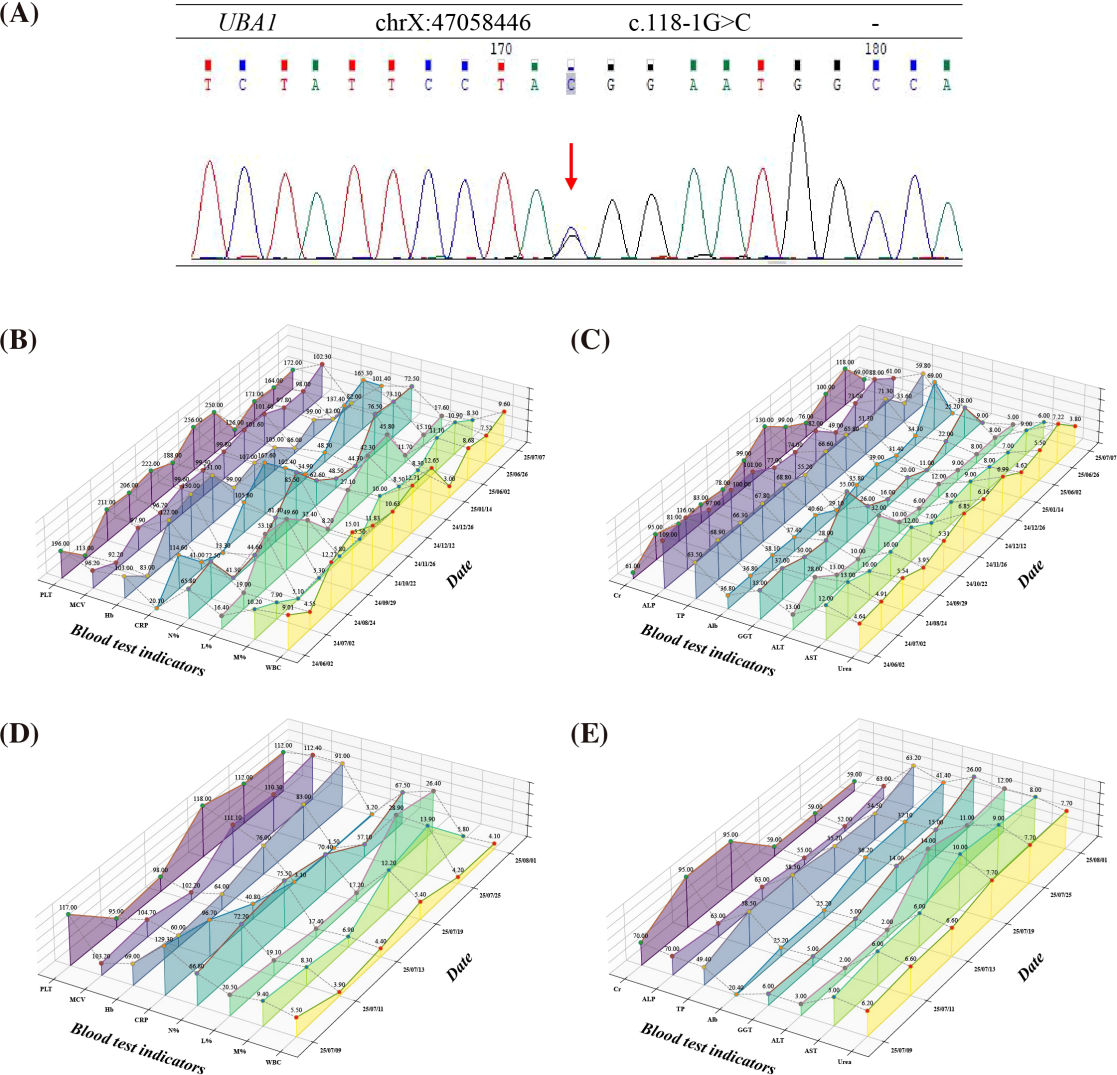
Serial haematological, biochemical, and inflammatory markers before and during admission. **(A)** Sanger sequencing analysis illustrating the point c.118-1G>C mutation, which is located at the intron–exon splice junction of the affected gene, indicating a possible splicing abnormality. **(B, C)** Longitudinal laboratory data collected at local hospitals (June 2024 – July 2025) showing temporal changes in key hematologic indices (PLT, Hb, MCV, WBC, etc.), inflammatory marker CRP and Serum biochemical indicators (Alb, TP, ALT, etc.). **(D, E)** Dynamic changes in blood test indicators after patient admission to our hospital. Upon presentation at the local hospital, the patient exhibited persistent or recurrent inflammatory activity, along with progressive hematological abnormalities, indicating chronic multi-organ involvement. These findings underscore the close association between VEXAS syndrome and systemic inflammation, as well as organ dysfunction. On admission, the patient displayed signs of active inflammation (elevated CRP), hepatic involvement (increased ALT/AST levels and decreased albumin), and hematological abnormalities (reduced PLT and Hb). Following corticosteroid therapy, there was a marked reduction in CRP levels, with gradual normalization of liver function and hematological parameters. These findings suggest that systemic inflammation was effectively controlled, resulting in notable improvement in the patient’s clinical condition. Each data point represents an individual measurement, with shaded areas or dashed lines indicating the normal reference range. Abbreviations: PLT: platelet count (normal range [NR] 125-350), MCV: mean corpuscular volume (NR 82-100), Hb: haemoglobin (NR 130-175), C-reactive protein (CRP, NR 0-5), N%: neutrophil percentage (NR 40-75%), L%: lymphocyte percentage (NR 20-50%), M%: monocyte percentage (NR 3-10%), WBC: white blood cell count (NR 3.5-9.5), Alb: albumin (NR 40-55), TP: total protein (NR 65-85), ALT: alanine aminotransferase (NR 9-50), AST: aspartate aminotransferase (NR 15-40), GGT: γ-glutamyltransferase (NR 10-60), ALP: alkaline phosphatase (NR 45-125), Cr: creatinine (NR 57-111), and Urea (NR 3.6-9.5).

In June 2024, a 77-year-old Chinese man presented to a local hospital with with exertional fever (maximum temperature 38.6°C), accompanied by fatigue, myalgias, and a red patchy rash on the neck. He had no chills, rigors, chest tightness, or chest pain. Initial laboratory tests revealed mild anemia (hemoglobin 104 g/L, mean corpuscular volume 96 fL), leukocytosis (white blood cell count 14.03 × 10^9^/L with neutrophils 53.6%, lymphocytes 39.3%), thrombocytosis (platelet count 419 × 10^9^/L), and elevated C-reactive protein (CRP 30.7 mg/L). Chest CT revealed scattered fibrotic foci in both lungs, localized emphysema in the upper lobes, segmental atelectasis in the lower lobes, aortic wall calcification, and enlargement of bilateral axillary and inguinal lymph nodes. Holter monitoring showed frequent multifocal atrial premature beats and occasional ventricular premature beats. Over the next year, he was hospitalized multiple times for similar episodes. Empiric antibiotics (e.g., moxifloxacin) were ineffective, and antipyretics (e.g., acetaminophen) provided only transient relief. Combined therapy with prednisone (40 mg/day) improved symptoms, but they would recur with exertion after the steroid was tapered. Serial laboratory markers (**Figures 1B, C**) and imaging studies continued to show persistent inflammation and stable lung fibrosis, suggesting a possible autoimmune process. Therefore, a maintenance dose of prednisone 10 mg/day was instituted. Two months before the current admission, the patient developed fever with sudden chest tightness, which resolved by sublingual nitroglycerin. Three days before admission, the fever recurred with severe chest tightness and dyspnea. An electrocardiogram showed atrial fibrillation and ST-T changes in leads II, aVF, and V1–V6. The patient came to our hospital for cardiac evaluation; exertion during transfer aggravated his symptoms. Given the significantly elevated CRP, he was referred from the outpatient clinic to the infectious diseases department for hospitalization. His past medical history included hypertension (well controlled on medication) and a transurethral resection of the prostate for benign prostatic hyperplasia.

**Figure 2:**
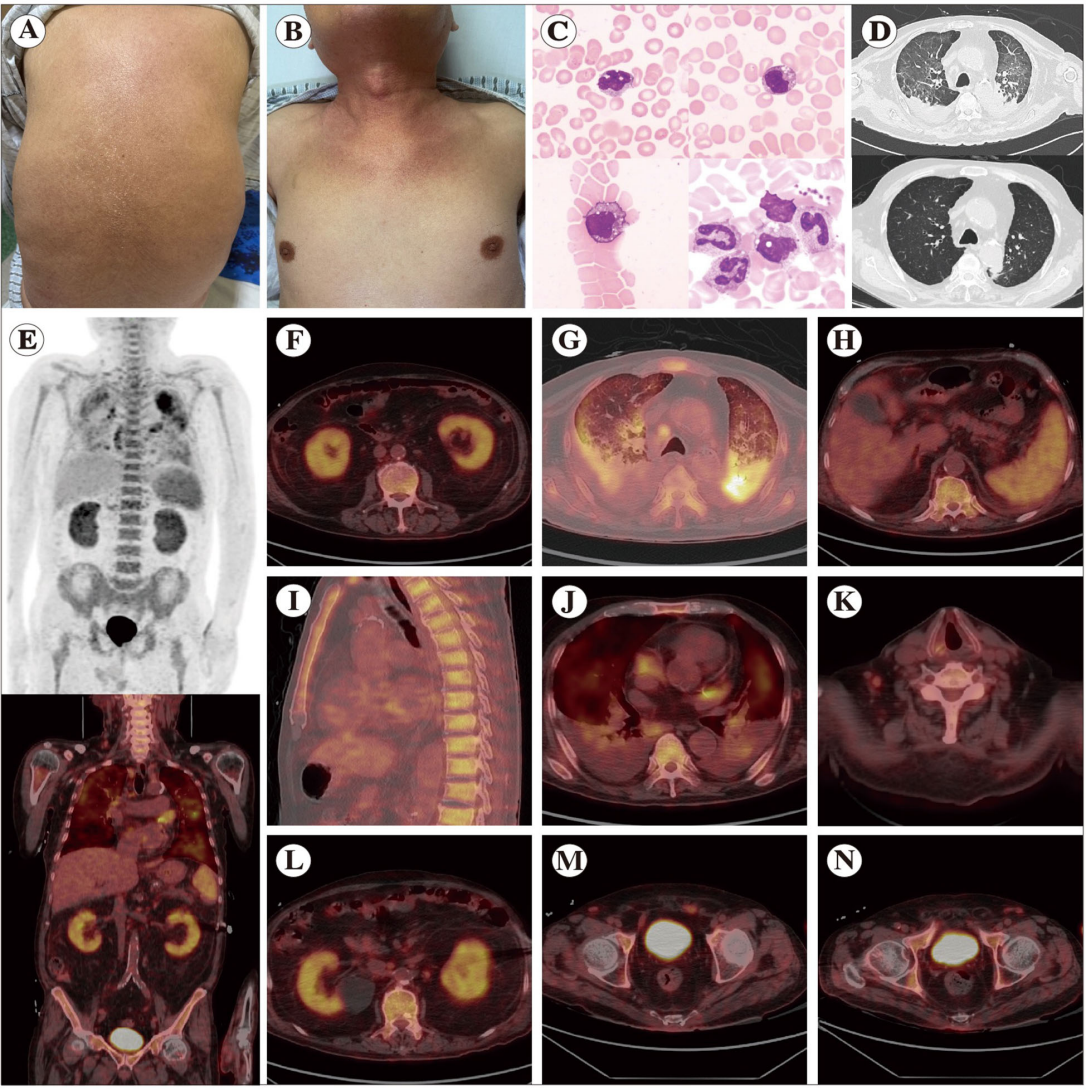
Skin, marrow vacuoles, and FDG-PET hypermetabolism in the patient. **(A)** Before corticosteroid therapy, the back shows xerosis with accentuated skin markings and surface desquamation and **(B)** the upper chest and neck display erythematous patchy plaques with accentuated skin markings. **(C)** Bone-marrow cytology shows a reduced nucleated cell count with granulocytic hyperplasia dominated by promyelocytic and earlier precursors; myeloid precursors exhibit the characteristic cytoplasmic vacuolation of VEXAS. **(D)** Chest CT before (top) and after (bottom) corticosteroid therapy: at presentation, bilateral patchy, ill-defined, faintly increased attenuation; after treatment, marked resolution of pleural effusions and inflammatory infiltrates with reduction of fibrotic foci. **(E, I)** Whole-body 18F-FDG PET-CT demonstrating diffuse, marrow-predominant hypermetabolism (SUVmax 5.0); additional increased uptake in the **(F)** kidneys (SUVmax 4.5), **(G)** lungs (SUVmax 7.7), **(H)** spleen (SUVmax 4.2), **(J)** both atria (SUVmax 5.37), **(K)** cervical/supraclavicular lymph nodes (SUVmax 3.9), and **(L–N)** retroperitoneal and bilateral inguinal nodes (SUVmax 2.5). These images collectively illustrate the patient’s key diagnostic features, including skin manifestations that highlight autoinflammatory involvement, which is essential for distinguishing VEXAS syndrome from infections or allergic conditions. The imaging findings also reveal widespread multi-organ inflammation, playing a critical role in ruling out infection, malignancy, or other forms of vasculitis.

On admission, physical examination showed dry, desquamative skin over the body (**Figures 2A, B**), decreased breath sounds bilaterally, mild edema of the face and lower limbs, and collapse of the nasal cartilage. Three hours after admission, the patient’s chest tightness worsened markedly; swelling and pruritus of the auricles were observed, and his pulse oximetry saturation rapidly dropped to 80%. The hypoxia improved after 30 minutes of oxygen therapy via face mask at 5 L/min. Admission laboratory tests (**Figures 1D, E**; **Table 1**) showed macrocytic anemia(hemoglobin 69 g/L, MCV 103.2 fL), thrombocytopenia (platelet count 117 × 10^9^/L), hypoalbuminemia (albumin 20.4 g/L), and markedly elevated inflammatory markers (CRP 129.3 mg/L, erythrocyte sedimentation rate 110 mm/h, interleukin-6 342.19 pg/mL, ferritin 1961.89 ng/mL; other cytokines were normal). Procalcitonin (0.68 ng/mL), prothrombin time (28.0 s) and D-dimer (384 μg/L) were mildly elevated. An emergency chest CT showed multifocal inflammatory infiltrates in both lungs (**Figure 2D**), bilateral pleural effusions with atelectasis of the lower lobes, and calcified plaques on the aortic and coronary artery walls.

**Table 1:** Key blood test indicators before and after treatment in the patient.

Blood test indicators	Prior to admission for treatment	Discharged after treatment	Normal range
CRP	129.3 mg/L	<0.2 mg/L	0.0-5.0 mg/L
Fer	1961.89 ng/ml	642.9 ng/ml	27.0-375.0 mg/L
IL-6	342.19 pg/ml	4.68 pg/ml	≤5.40 pg/ml
PCT	0.68 ng/ml	0.1 ng/ml	0.00-0.50 ng/ml
HBP	53.76 ng/ml	25.7 ng/ml	<15.00 ng/ml
ESR	110 mm/h	3 mm/h	0–15 mm/h
WBC	5.5×10^9^/L	5×10^9^/L	3.5-9.5×10^9^/L
N%	66.8%	51.7%	40.0-75.0%
L%	20.5%	42%	20.0-50.0%
Hb	69 g/L	108 g/L	130–175 g/L
MCV	103.2 fL	102.2 fL	82.0-100.0 fL
MCHC	315 g/L	329 g/L	316–354 g/L
PLT	117×10^9^/L	150×10^9^/L	125-350×10^9^/L
Alb	20.4 g/L	34.3 g/L	40.0-55.0 g/L
D-dimer	384 μg/L	222 μg/L	0-243 μg/L
PT	28.0 s	11.9 s	9.4-12.5 s
Troponin I	1.08 ng/ml	0.026 ng/ml	0-0.026 ng/ml
NT-proBNP	5109 pg/ml	366 pg/ml	0–450 pg/ml

CRP, C-reactive protein; Fer, ferritin; IL-6, interleukin-6; PCT, procalcitonin; HBP, heparin-binding protein; ESR, erythrocyte sedimentation rate; WBC, white blood cell count; N%, neutrophil percentage; L%, lymphocyte percentage; Hb, hemoglobin; MCV, mean corpuscular volume; MCHC, mean corpuscular hemoglobin concentration; PLT, platelet count; Alb, albumin; PT, prothrombin time; NT-proBNP, N-terminal pro-brain natriuretic peptide.

The next day, whole-body 18F-FDG PET-CT showed diffuse hypermetabolism in the bone marrow (**Figure 2E**). with increased metabolic activity in the spleen, lungs, kidneys, atria, and lymph nodes, but no evidennce of tumor (**Figures 2F–N**). Suspecting a small-vessel vasculitis, we empirically administered methylprednisolone 80 mg/day with intravenous immunoglobulin (IVIG) 20 g/400 mL/day for anti-inflammatory therapy, and rivaroxaban 10 mg/day for anticoagulation. Subsequently, NGS of the blood for pathogens was negative. Accordingly, we adjusted the anti-infective regimen to oral trimethoprim-sulfamethoxazole combined with micafungin for prophylaxis against pulmonary fungal infection and secondary bacterial infection. Autoimmune serologies were negative (including antineutrophil cytoplasmic antibodies, anti-endothelial cell antibodies, and anti–double-stranded DNA; antinuclear antibody was borderline at 1:100). Urinary κ and λ light chains were within normal range. A 24-hour Holter monitor recorded a total of 7,662 atrial premature beats. To further clarify the etiology, we performed whole-exome sequencing.

After two weeks of tapered steroid therapy, the patient’s symptoms improved and abnormal laboratory values gradually normalized (**Table 1**, **2**). Whole-exome sequencing identified a somatic UBA1 splice-site mutation (c.118-1G>C; ClinVar VCV001298353.3, Pathogenic) (**Figure 1A**), suggesting the possibility of VEXAS syndrome. Given reports of hematopoietic abnormalities in VEXAS, we performed a bone marrow aspiration. Bone marrow pathology (**Figure 2C**) showed characteristic cytoplasmic vacuolization in a subset of myeloid precursor cells, with no dysplasia in the erythroid or myeloid lineages, consistent with VEXAS syndrome marrow findings. The diagnosis of VEXAS syndrome was finally confirmed. As the patient’s condition had stabilized, we continued glucocorticoid monotherapy. After a period of treatment, Holter monitoring showed the atrial premature beats had decreased to 564, a significant reduction. High-resolution chest CT demonstrated marked improvement of the pulmonary inflammatory lesions and resolution of the pleural effusions (**Figure 2D**). No significantly enlarged lymph nodes were detected in the bilateral neck, axillae, or groin.

**Table 2:** Clinical features and imaging findings before and after treatment in the patient.

	Prior to admission for treatment	Discharged after treatment
Vital signs	Temperature of 40.1°C, Heart rate 150 bpm, Respiratory rate 35, BP 108/70 mmHg, O_2_ saturation 80% on room air	Temperature of 36.8°C, Heart rate 68 bpm, Respiratory rate 16, BP 121/59 mmHg, O_2_ saturation 98% on room air
Edema	Facial and bilateral lower limb edema	Symptom improvement
Skin Involvement	Generalized skin desquamation and dryness	Symptom improvement
Chondritis	Nasal and auricular chondritis	Symptom improvement
Pulmonary Involvement	● Chest tightness and dyspnea ● Multiple inflammatory lesions in both lungs, bilateral pleural effusion with atelectasis of both lower lobes	● Symptom improvement ● Significant resolution of pulmonary inflammatory lesions; bilateral pleural effusion absorbed
Cardiac Involvement	● Atrial fibrillation with chest pain ● Elevated troponin and NT-proBNP ● Holter: 7662 supraventricular premature beats ● 18F-FDG PET-CT scan indicates increased metabolism in both atria.	● Atrial fibrillation resolved, chest pain relieved ● Relevant blood test indicators have returned to normal levels ● Holter: Supraventricular premature beats significantly improved (564 supraventricular premature beats)
Bone Marrow Involvement	18F-FDG PET-CT showed diffusely increased bone marrow metabolism	Close follow-up; repeat 18F-FDG PET-CT at six months after discharge to assess bone marrow involvement
Splenomegaly	18F-FDG PET-CT showed splenomegaly with increased metabolic activity	Ultrasound shows no significant enlargement of the spleen
Lymph Nodes	18F-FDG PET-CT showed multiple enlarged lymph nodes with increased metabolism in bilateral cervical, supraclavicular, mediastinal, hilar, and inguinal regions	Ultrasound showed no obvious enlarged lymph nodes in the cervical, axillary, or inguinal regions
Treatment	Methylprednisolone 80 mg/day + Intravenous immunoglobulin (IVIG) 20 g/400 mL/day, intravenous infusion	Discharge medication: Oral methylprednisolone tablets 12 mg/day

bpm, beats per minute; BP: blood pressure; NT-proBNP: N-terminal pro-brain natriuretic peptide; 18F-FDG PET-CT: 18F-fluorodeoxyglucose positron emission tomograph–computed tomography

## Discussion

VEXAS syndrome is an autoinflammatory disease caused by somatic mutations in the UBA1 gene, predominantly affecting older men. It is characterized by recurrent fever, multi-organ involvement, vasculitis, and progression to hematologic malignancy (1, 7). UBA1 encodes ubiquitin-like modifier activating enzyme 1 (8), which plays a key role in protein ubiquitylation—a post-translational modification that regulates protein degradation, cell signaling, and innate immune responses. Pathogenic variants result in loss of function, predominantly affecting the cytoplasmic UBA1b isoform, leading to dysregulated ubiquitination, activation of innate immune pathways (such as NF-κB and interferon signaling), and systemic inflammation (9) This myeloid-driven pathological process arises from a somatic mutation in hematopoietic stem cells that promotes clonal expansion of mutant cells. It not only triggers an inflammatory cascade but also produces hematologic abnormalities such as macrocytic anemia and cytoplasmic vacuolization in precursor cells (10). In our patient, the acute decline in pulmonary function and the onset of arrhythmias were likely due to cytokine-mediated endothelial injury and tissue infiltration by activated myeloid cells, highlighting the role of an autoinflammatory cascade in organ-specific pathology.

Notably, The UBA1 c.118-1G>C splice-site mutation found in our patient is extremely rare in VEXAS syndrome. In previously reported cases, the p.Met41 variant has predominated. The p.Met41 mutation disrupts the translation start codon for UBA1b in exon 3, resulting in reduced expression of the cytoplasmic isoform and compensatory reliance on the nuclear UBA1a isoform. In contrast, splice-site mutations such as c.118-1G>C (located at the intron 2–exon 3 junction) cause aberrant splicing that may lead to exon 3 skipping or introduce a premature termination codon (11–13). Structurally, UBA1 consists of an inactive adenylation domain (IAD), an active adenylation domain (AAD), and a ubiquitin-fold domain. The p.Met41 variant impairs AAD activation, causing the ubiquitination efficiency of certain mutants (e.g., p.Ser56) to decrease in a temperature-dependent manner. By contrast, a splice-site mutation like c.118-1G>C may broadly disrupt protein folding or alter isoform ratios (11, 14). Mechanistically, both types of mutations trigger an unfolded protein response and hyperactivation of innate immunity. However, splice-site variants may exhibit differing penetrance or a milder phenotype — for example, a case with c.118-1G>C achieved complete remission with a hypomethylating agent, whereas patients with p.Met41 mutations tend to show higher glucocorticoid dependence (10). The c.118-1G>C mutation identified in our patient has not been previously reported in China. Its identification expands the phenotypic spectrum of VEXAS associated with this variant and suggests that future clinical studies and screening should consider a wider range of loss-of-function UBA1 mutations.

This patient exhibited interstitial pneumonia, skin rash, auricular and nasal chondritis (relapsing polychondritis), and cardiac involvement, which align well with the commonly reported features of VEXAS syndrome (1, 7, 15). Pulmonary infiltrates or inflammation are among the most frequent findings; both Beck et al. (1) and a French multicenter cohort (15) have indicated that the majority of patients have lung involvement. Auricular or nasal chondritis (relapsing polychondritis) is also typical; in Beck’s series (1), 64% of patients had auricular or nasal chondritis, and Ferrada et al. (7) found a similarly high prevalence of chondritis and skin lesions in VEXAS patients with relapsing polychondritis. Skin involvement is very common in VEXAS, often presenting as neutrophilic dermatoses; a French registry reported that 83% of patients had skin lesions (15). Unlike the typical erythematous plaques or papules, our patient’s skin showed xerosis-like “ichthyosiform” changes with extensive desquamation. By contrast, cardiac involvement is relatively rare, with only a few series noting pericarditis, myocarditis, or arrhythmias. Van der Made et al. (16) reported a few patients with “cardiac involvement.” Recent studies suggest that about 4–33% of VEXAS patients may experience cardiac complications (such as pericarditis or myocarditis) (17, 18). In our case, the patient had frequent atrial premature beats, atrial fibrillation, and ST-segment changes, suggesting cardiac inflammation. Although cardiac involvement is uncommon in VEXAS reports, our findings are consistent with these observations. Compared to previous reports, our patient’s multi-organ involvement and complications progressed relatively rapidly. Many VEXAS patients initially present with recurrent fever or chondritis, whereas in our case the initial presentation was fever with pulmonary inflammation, and chondritis appeared only after acute disease exacerbation. Overall, the lung, skin, and cartilage involvement in our patient are typical of VEXAS, but the cardiac involvement and the acute, life-threatening pulmonary deterioration are relatively uncommon, emphasizing the clinical heterogeneity of VEXAS syndrome.

The diagnostic pathway in this case was also noteworthy. In suspected VEXAS cases, experts recommend UBA1-targeted sequencing or whole-exome sequencing to identify somatic mutations, which are often detectable at high frequency in peripheral blood (1, 2, 19, 20). In our case, whole-exome sequencing revealed the c.118-1G>C somatic mutation, confirming the diagnosis of VEXAS syndrome. The characteristic vacuolization observed on bone marrow morphology further supported the diagnosis (21). In contrast, diagnosing VEXAS based solely on clinical features is challenging and prone to error. Our patient was initially misdiagnosed with small-vessel vasculitis; after NGS and PET-CT excluded infection and malignancy, we pursued empirical therapy for vasculitis, but only genetic testing ultimately revealed the underlying cause. This underscores the value of modern multidisciplinary collaboration and advanced diagnostic techniques in the early identification of VEXAS syndrome (22, 23).

In terms of treatment, we administered high-dose glucocorticoids with IVIG, achieving rapid control of inflammation. Similar to reports where IVIG has been combined with glucocorticoids in refractory VEXAS cases to enhance anti-inflammatory effects and mitigate steroid reliance (24). Beck et al. and GeneReviews both note that glucocorticoids are the first-line treatment of choice for VEXAS, often requiring high doses to suppress inflammation (1, 2). Our patient had a marked improvement on prednisone 40 mg/day, but relapsed after tapering, consistent with the reported difficulty in maintaining remission and the high steroid dependence. Magnol et al. (25) reported a VEXAS patient who, after failing multiple biologic agents, achieved symptom resolution with IVIG plus an IL-17 inhibitor, suggesting that when conventional therapies are ineffective, IVIG can serve as an adjunctive treatment. The IVIG used in our case may also have accelerated the resolution of inflammation. Additionally, given the predisposition of VEXAS patients to infection (especially pulmonary infection), we employed broad-spectrum antimicrobial therapy during the acute phase and recommend prophylactic antibiotics thereafter, consistent with recent reports emphasizing the high infection risk in VEXAS (5). Although some studies report that JAK inhibitors (e.g., ruxolitinib), anti-IL-6 agents (tocilizumab), or anakinra can alleviate symptoms (4, 17, 26, 27), we have not yet used these therapies. Finally, long-term follow-up remains essential.

## Conclusion

This case highlights several unique aspects of VEXAS syndrome. In our clinical management, we focused on the patient’s rapidly progressive pulmonary interstitial pneumonia and used NGS and PET-CT to exclude infection and hematologic malignancy, concentrating on empirical treatment of presumptive small-vessel vasculitis to quickly halt disease progression. Meanwhile, whole-exome sequencing ultimately identified the underlying cause. In summary, this case report contributes to improving clinical awareness of the diverse mutations and manifestations of VEXAS syndrome and provides a reference for early recognition and treatment of similar patients.

## Data Availability

The original contributions presented in the study are included in the article/supplementary material. Further inquiries can be directed to the corresponding authors.
